# Dietary supplementation with inosine-5′-monophosphate improves the functional, energetic, and antioxidant status of liver and muscle growth in pigs

**DOI:** 10.1038/s41598-021-04023-y

**Published:** 2022-01-10

**Authors:** Lucas P. Bonagurio, Alice E. Murakami, Camila A. Moreira, Jurandir F. Comar, Paulo C. Pozza

**Affiliations:** 1 Department of Animal Sciences, State University of Maringá, Maringá, PR Brazil; 2 Department of Biochemistry, State University of Maringá, Maringá, PR Brazil

**Keywords:** Animal physiology, Metabolic pathways

## Abstract

Inosine 5′-monophosphate (5′-IMP) is an essential nucleotide for de novo nucleotide biosynthesis and metabolism of energy, proteins, and antioxidants. Nucleotides are conditionally essential, as they cannot be produced sufficiently rapidly to meet the needs of the body in situations of oxidative stress or rapid muscle growth. A deficient intake of nucleotides can result in decreased ATP and GTP synthesis and impaired metabolism. We demonstrated that supplementation of finishing pig diets with 5′-IMP reduces the relative weight of the liver, and increases oxygen consumption during mitochondrial respiration without changing the ADP/O ratio, indicating an increase in the respiratory efficiency of liver mitochondria. We also observed a reduction in liver lipid peroxidation and an increase in muscle creatine. Moreover, 5′IMP supplementation increases slaughter weight, lean meat yield, sarcomere length, and backfat thickness in finishing barrows, demonstrating influence on protein metabolism. We suggest that 5′-IMP supplementation increase the mitochondrial respiratory capacity when the liver metabolic activity is stimulated, enhances antioxidant defense, and promotes muscle growth in finishing barrows.

## Introduction

Oxidative phosphorylation, the main pathway for the synthesis of energy in the form of ATP, takes place in the inner membrane of mitochondria via the electron transport chain^1,2^. Complexes I, III, IV, and V (ATP synthetase) are essential for oxidative phosphorylation. The electrons of complexes I and III react with oxygen molecules to form superoxide radicals, which are responsible for the generation of reactive oxygen species (ROS). In excess, ROS are known to cause damage to mitochondrial DNA^3^.

Mitochondrial DNA is susceptible to oxidation because of the frequent exposure to ROS produced during oxidative phosphorylation and the deficiency of antioxidant molecules in the inner membrane of mitochondria^4,5^. Thus, the presence of antioxidant molecules that can be converted into ADP and subsequently into ATP may promote benefits to oxidative phosphorylation and help repair ROS-induced damage to mitochondrial DNA. An important molecule with such characteristics is the nucleotide inosine 5′-monophosphate (5′-IMP). 5′-IMP can be converted to ADP or GDP and, subsequently, to ATP or GTP^6,7^. There is increasing evidence that 5′-IMP plays an important role in DNA repair, as observed in the deamination of adenosine to inosine, which depends on the actions of the adenosine deaminase family of enzymes on RNA^8^. Another important function of 5′-IMP is in mTORC1 complex that serves as a link between energy, nutrient levels and anabolic processes^9^.

Given the importance of 5′-IMP to metabolism, it is possible that dietary supplementation of finishing barrows with 5′-IMP may influence several metabolisms. We hypothesized that diets supplemented with 5′-IMP may promote beneficial additive effects on antioxidant, protein and energy metabolism of finishing barrows. This study aimed to investigate the effects of low- (3200 kcal ME/kg) and high-energy (3300 kcal ME/kg) diets without 5′-IMP with those of low-energy diets (3200 kcal ME/kg) containing varying levels of 5′-IMP on liver antioxidant status, respiratory activity of isolated liver mitochondria, plasma concentrations of alanine aminotransferase (ALT) and aspartate aminotransferase (AST), plasma and muscle levels of creatine, carcass traits, and sarcomere length in *longissimus lumborum* muscle in finishing pigs (75–100 kg).

## Results

### Relative weight of the liver

Tables 1 and 2 describe the effects of control (negative and positive) and 5′-IMP-supplemented diets on relative weight of liver. Dietary supplementation with 5′-IMP influenced the relative weight of the liver (*P* < 0.05) (Table 3). Orthogonal contrast analysis of relative liver weights showed that supplementation with 0.150 and 0.200% 5′-IMP decreased (*P* ≤ 0.05) the variable compared with CN and PC diets. Furthermore, we observed that the relative weight of the liver decreased linearly (*P* < 0.001) with increasing 5′-IMP supplementation levels (Fig. 2A).

**Table 1:** Oxygen consumption at different states of respiration in mitochondria isolated from the liver of 75–100 kg barrows fed diets containing different levels of inosine-5-monophosphate (5′-IMP) and metabolizable energy (ME), using succinate or α-ketoglutarate as respiratory substrate and stimulated by adenosine monophosphate (ADP).

Item	Liver relative weight, %	Succinate					α-Ketoglutarate				
		Basal^c^	III^d^	IV^e^	RC^f^	ADP/O^g^	Basal	III	IV	RC	ADP/O
NC^a^	1.708	14.47	43.55	16.69	2.68	2.00	5.44	28.09	11.60	2.23	2.57
PC^b^	1.753	13.92	45.19	15.99	2.89	1.84	5.17	25.99	10.25	2.56	2.21
0.050%	1.773	13.00	49.10	15.02	3.28	1.73	6.83	23.80	10.27	2.48	2.42
0.100%	1.758	14.75	47.75	14.79	3.22	1.97	5.73	25.59	10.34	2.44	2.26
0.150%	1.601	12.98	44.65	16.62	2.70	1.74	5.69	27.30	11.78	2.42	2.21
0.200%	1.560	14.68	69.20	18.76	3.82	2.03	5.26	34.48	11.90	2.93	2.36
Mean	1.687	13.97	49.90	16.31	3.09	1.89	5.68	27.54	10.99	2.51	2.33
SD	0.132	2.132	11.521	2.996	0.656	0.304	1.016	4.407	1.630	0.384	0.348
SEM	0.047	0.360	1.947	0.506	0.111	0.051	0.172	0.745	0.276	0.065	0.059
NC × PC	0.336	0.671	0.721	0.683	0.517	0.349	0.625	0.295	0.206	0.161	0.116
NC × 0.050%	0.152	0.255	0.232	0.336	0.072	0.119	0.015	0.038	0.216	0.290	0.509
NC × 0.100%	0.216	0.823	0.363	0.274	0.105	0.883	0.612	0.211	0.240	0.347	0.152
NC × 0.150%	0.039	0.248	0.810	0.967	0.947	0.142	0.647	0.690	0.829	0.413	0.102
NC × 0.200%	0.004	0.865	< 0.001	0.234	0.001	0.875	0.738	0.003	0.740	0.005	0.347
PC × 0.050%	0.560	0.671	0.396	0.683	0.236	0.565	0.005	0.273	0.975	0.717	0.346
PC × 0.100%	0.837	0.471	0.577	0.576	0.316	0.424	0.333	0.828	0.917	0.635	0.825
PC × 0.150%	0.004	0.518	0.907	0.713	0.560	0.598	0.347	0.512	0.102	0.544	0.997
PC × 0.200%	< 0.001	0.553	< 0.001	0.114	0.007	0.279	0.878	< 0.001	0.079	0.113	0.508
Linear	< 0.001	0.476	< 0.001	0.503	0.050	0.257	0.063	0.002	0.315	0.008	0.225
Quadratic	0.214	0.734	0.020	0.272	0.056	0.215	0.022	< 0.001	0.151	0.325	0.244

^a^Negative control (0.00% 5′-IMP and 3200 kcal ME/kg).

^b^Positive control (0.00% 5′-IMP and 3300 kcal ME/kg).

^c^Oxygen consumption before ADP addition (substrate or basal).

^d^Oxygen consumption shortly after ADP addition (state III).

^e^Oxygen consumption after stopping ADP stimulation (state IV).

^f^Respiratory control (RC) calculated by the relationship between states III/IV.

^g^ADP/O ratio determined as proposed by Chance and Williams (1955).

**Table 2:** Fitted regression equations for the studied parameters as a function of dietary inosine-5′-monophosphate (5′-IMP) level.

Item	Quadratic equations	*R*^2^	IP^k^	5′-IMP	PC^l^
Basal (k)^a^	Ẏ = 5.70 + 14.41 × 5′-IMP − 87.08 × 5′-IMP^2^	0.82	6.27	0.083	− 0.030
State III(s)^b^	Ẏ = 48.03 − 113.55 × 5′-IMP + 1036.48 × 5′-IMP^2^	0.68	44.91	0.055	0.056
State III(k)^c^	Ẏ = 27.86 − 98.05 × 5′-IMP + 653.03 × 5′-IMP^2^	0.93	24.18	0.075	0.074
DPPH, %^d^	Ẏ = 30.31 − 143.94 × 5′-IMP + 710.98 × 5′-IMP^2^	0.90	23.02	0.101	0.107
MDA, mg/dL^e^	Ẏ = 1.554 − 5.427 × 5′-IMP + 27.907 × 5′-IMP^2^	0.60	1.29	0.097	0.199
Slaughter weight, kg	Ẏ = 97.27 + 41.12 × 5′-IMP − 149.44 × 5′-IMP^2^	0.65	100.09	0.138	0.017
LMY, %^f^	Ẏ = 58.02 + 10.43 × 5′-IMP − 35.26 × 5′-IMP^2^	0.91	58.79	0.148	0.095
BT, cm^g^	Ẏ = 1.05 + 5.85 × 5′-IMP − 27.02 × 5′-IMP^2^	0.53	1.37	0.108	0.101
**Linear equations**					
Relative liver weight	Ẏ = 1.744 − 0.936 × 5′-IMP	0.60	–	–	0.022
RC (s)^h^	Ẏ = 2.82 − 3.43 × 5′-IMP	0.84	–	–	0.084
RC (k)^i^	Ẏ = 2.32 + 2.68 × 5′-IMP	0.43	–	–	0.091
Creatine mg/dL	Ẏ = 5.68 + 2.82 × 5′-IMP	0.67	–	–	− 0.124
SL45min, µm^j^	Ẏ = 1.728 + 0.204 × 5′-IMP	0.67	–	–	− 0.108

^a^Basal state respiration rate in liver mitochondria incubated with α-ketoglutarate.

^b^State III respiration rate in liver mitochondria incubated with succinate, stimulated by ADP.

^c^State III respiration rate in liver mitochondria incubated with α-ketoglutarate and stimulated by ADP.

^d^Free radical scavenging assay.

^e^Malondialdehyde concentration.

^f^Lean meat yield.

^g^Backfat thickness.

^h^Respiratory control of liver mitochondria incubated with succinate.

^i^Respiratory control of liver mitochondria incubated with α-ketoglutarate.

^j^Sarcomere length at 45 min after slaughter.

^k^Inflection point.

^l^Positive control diet (0.00% 5′-IMP and 3300 kcal ME/kg).

**Table 3:** Carcass traits and sarcomere length in *longissimus lumborum* muscle of 75–100 kg barrows fed diets containing different levels of inosine-5-monophosphate (5′-IMP) and metabolizable energy (ME).

Item	Slaughter weight, kg	LMY^c^, %	LLD^f^, cm	SL45 min^e^, µm	SL24 h^f^, µm	BT^g^, cm
NC^a^	97.03	58.05	6.03	1.729	1.693	0.97
PC^b^	97.94	58.69	6.08	1.706	1.673	1.44
0.050%	98.24	58.49	6.28	1.731	1.695	1.44
0.100%	98.48	58.65	6.06	1.743	1.688	1.31
0.150%	100.55	58.90	6.01	1.790	1.694	1.23
0.200%	98.56	58.69	5.91	1.750	1.680	1.21
Mean	98.47	58.56	6.07	1.740	1.690	1.27
SD	2.380	0.350	0.380	0.032	0.013	0.230
SEM	0.320	0.050	0.050	0.005	0.002	0.030
**Contrasts**						
NC × PC	0.353	< 0.001	0.627	0.045	0.001	0.002
NC × 0.050%	0.262	< 0.001	0.183	0.855	0.701	0.003
NC × 0.100%	0.156	< 0.001	0.763	0.169	0.386	0.009
NC × 0.150%	0.004	< 0.001	0.986	< 0.001	0.898	0.025
NC × 0.200%	0.155	< 0.001	0.751	0.052	0.025	0.098
PC × 0.050%	0.758	0.270	0.401	0.030	0.003	0.866
PC × 0.100%	0.556	0.835	0.951	0.013	0.010	0.590
PC × 0.150%	0.037	0.020	0.622	< 0.001	< 0.001	0.298
PC × 0.200%	0.589	0.590	0.451	0.002	0.224	0.168
**Statistical analysis**						
Linear	0.002	< 0.001	0.495	0.004	0.654	0.326
Quadratic	0.015	< 0.001	0.324	0.077	0.299	< 0.001

^a^Negative control (0.00% 5′-IMP and 3200 kcal ME/kg).

^b^Positive control (0.00% 5′-IMP and 3300 kcal ME/kg).

^c^Lean meat yield.

^d^*Longissimus lumborum* depth.

^e^Sarcomere length at 45 min after slaughter.

^f^Sarcomere length at 24 h after slaughter.

^g^Backfat thickness.

### Mitochondrial respiratory activity

**Figure 1:**
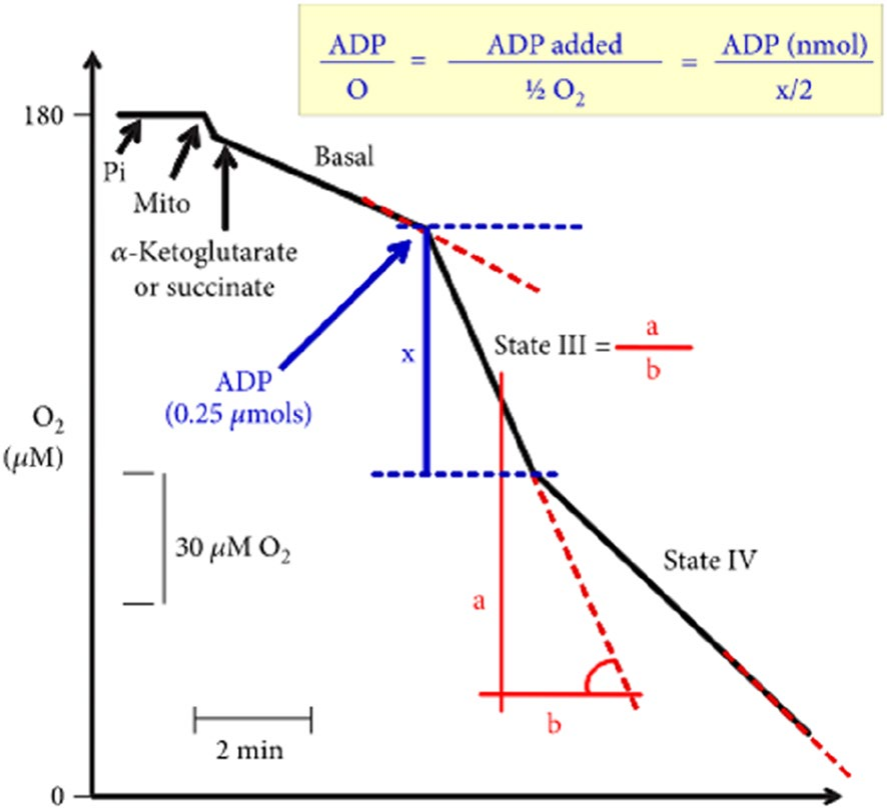
Experimental protocol and calculation procedures.

Tables 1 and 2 describe the effects of control (negative and positive) and 5′-IMP-supplemented diets on basal, III, and IV state respiration rates, respiratory control, and ADP/O ratio of liver mitochondria incubated with succinate or α-ketoglutarate. Figure 1 depicts the experimental approach used to assess the respiratory activity of liver mitochondria.

The basal respiration rate of liver mitochondria incubated with succinate did not differ significantly (*P* > 0.05) between treatments (Table 1). By contrast, in medium containing α-ketoglutarate, basal respiration rate was higher in mitochondria from pigs fed 0.050% 5′-IMP than in those from pigs fed negative control (*P* = 0.015) or positive control (*P* = 0.005) diets, but no differences were observed between control treatments (*P* = 0.625), as shown in Table 1.

**Figure 2:**
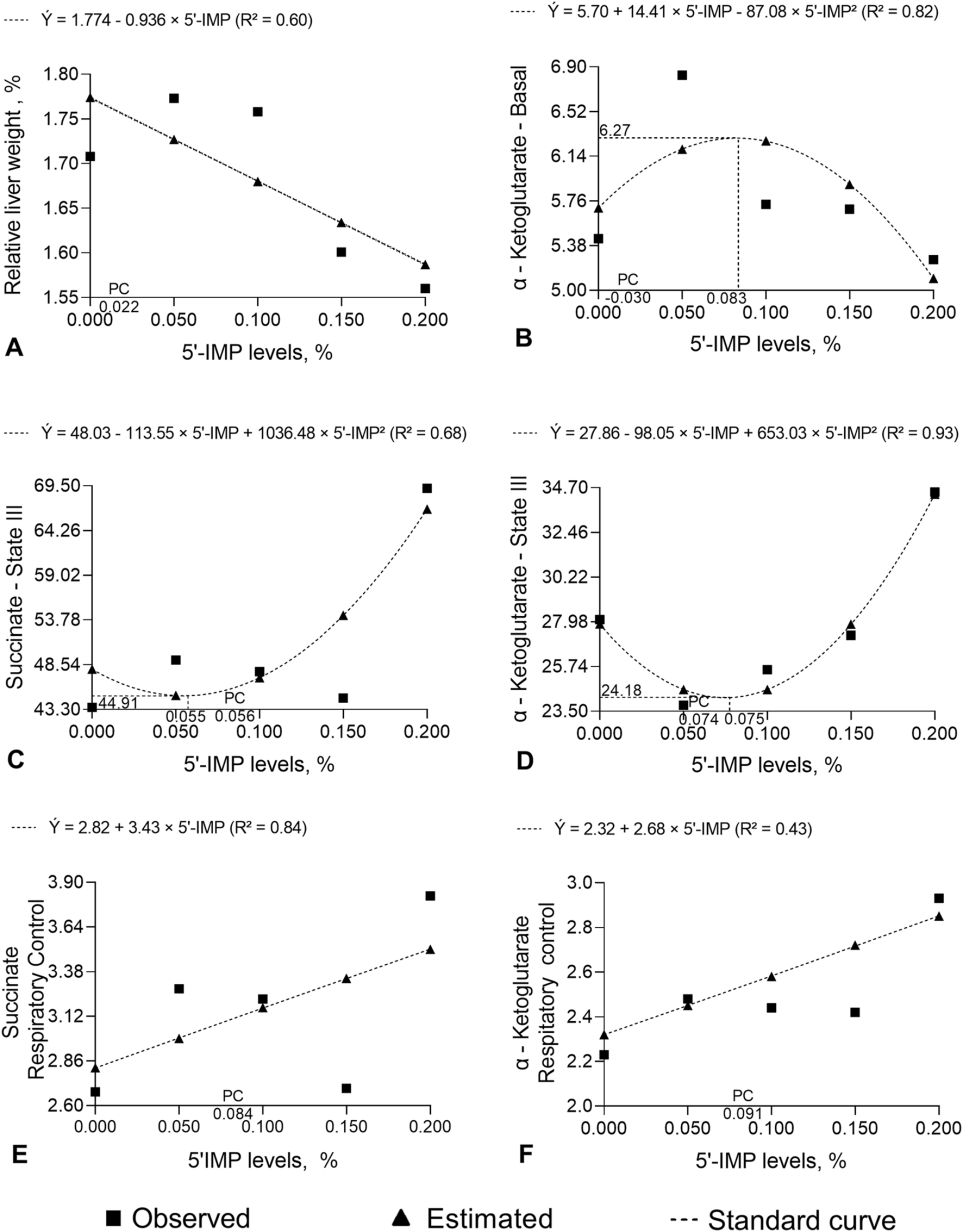
Relative liver weight and oxygen consumption at different states of respiration in mitochondria isolated from the liver of 75–100 kg barrows fed diets containing different levels of inosine-5′-monophosphate (5′-IMP) and a 100 kcal/kg lower metabolizable energy level than the positive control (high-energy) diet.

As depicted in Fig. 2B, the relationship between basal oxygen consumption in α-ketoglutarate medium and 5′-IMP level was quadratic (*P* = 0.022). The highest basal oxygen consumption (6.27) was estimated to be achieved by supplementation with 0.083% 5′-IMP.

No differences in state III respiration rates were observed between control treatments for mitochondria incubated with succinate (*P* = 0.721) or α-ketoglutarate (*P* = 0.295). However, the respiration rate of liver mitochondria from pigs supplemented with 0.200% 5′-IMP was higher than that of mitochondria from negative (*P* = 0.003) and positive (*P* < 0.001) control pigs (Table 1). Compared with the negative control diet, dietary 5′-IMP supplementation increased state III respiration rate (*P* = 0.038) in mitochondria incubated with α-ketoglutarate (Table 1).

The relationship between mitochondrial state III respiration rate in succinate medium and dietary 5′-IMP level was well explained by a quadratic model (*P* = 0.020) (Fig. 2C). The lowest respiration rate was estimated to be achieved by supplementation with 0.055% 5′-IMP and the highest rate (66.77%), with 0.200% 5′-IMP. Similarly, in α-ketoglutarate medium the state III respiration rate of liver mitochondria had a quadratic response (*P* < 0.001) to 5′-IMP supplementation (Fig. 2D). The lowest respiration rate was estimated to be achieved by supplementation with 0.075% 5′-IMP and the highest rate (34.37%), with 0.200% 5′-IMP.

No differences (*P* > 0.05) in state IV respiration rate were observed between treatments, regardless of incubation medium (Table 1). In succinate medium, respiration rate did not differ between negative and positive control diets (*P* = 0.161) but was higher (*P* < 0.05) with 0.050% and 0.200% 5′-IMP supplementation (Table 1). Only 0.200% 5′-IMP supplementation differed from the negative control diet (*P* = 0.005) with regard to state IV respiration rate in α-ketoglutarate medium.

As depicted in Fig. 2E,F, the respiratory control in succinate and in α-ketoglutarate medium increased linearly (*P* < 0.050) with 5′-IMP supplementation level. Thus, the highest respiratory control in the presence of succinate or α-ketoglutarate was also achieved with 0.200% 5′-IMP supplementation. ADP/O ratios did not differ between treatments (*P* > 0.05), regardless of incubation medium (Table 1).

### Liver antioxidant activity

**Figure 3:**
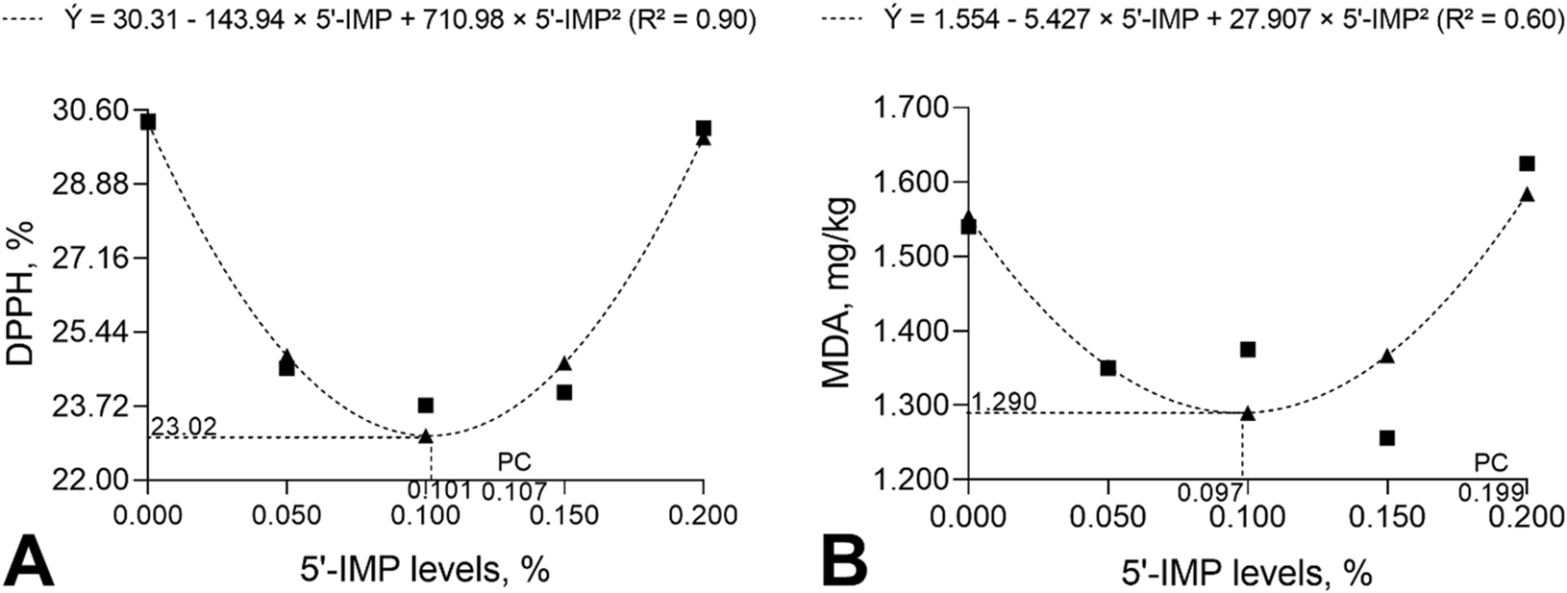
Free radical scavenging assay (**A**) and malondialdehyde (MDA) concentration (**B**) in the liver of 75–100 kg barrows fed diets containing different levels of inosine-5′-monophosphate (5′-IMP) and a 100 kcal/kg lower metabolizable energy level than the positive control (high-energy) diet.

Tables 2 and 4 show the differences between experimental diets and the effects of 5′-IMP supplementation level on 2,2-diphenyl-1-picrylhydrazyl radical (DPPH^•^) scavenging activity and malondialdehyde (MDA) concentration in liver tissues. The liver DPPH^•^ scavenging activity of pigs fed the negative control diet was higher (*P* < 0.001) than that of pigs fed the positive control diet (*P* ≤ 0.05) or diets supplemented with 0.050%, 0.100%, and 0.150% 5′-IMP. The positive control diet resulted in a lower (*P* < 0.001) DPPH^**·**^ scavenging activity than the 0.200% 5′-IMP-supplemented diet. DPPH^**·**^ activity was shown to have a quadratic relationship with 5′-IMP level (*P* < 0.001): the lowest activity (23.10%) was predicted to occur with 0.101% 5′-IMP supplementation (Fig. 3A).

**Table 4:** Free radical scavenging assay (DPPH %) and malondialdehyde concentration (MDA) in the liver of 75–100 kg barrows fed diets containing different levels of inosine-5-monophosphate (5′-IMP) and metabolizable energy (ME).

Item	DPPH, %	MDA, mg/kg
NC^a^	30.340	1.540
PC^b^	22.640	1.689
0.050%	24.600	1.350
0.100%	23.740	1.375
0.150%	24.040	1.256
0.200%	30.170	1.625
Mean	25.920	1.472
SD	3.854	0.194
SEM	0.556	0.028
**Contrasts**		
NC × PC	< 0.001	0.017
NC × 0.050%	< 0.001	0.003
NC × 0.100%	< 0.001	0.009
NC × 0.150%	< 0.001	< 0.001
NC × 0.200%	0.889	0.666
PC × 0.050%	0.108	< 0.001
PC × 0.100%	0.316	< 0.001
PC × 0.150%	0.242	< 0.001
PC × 0.200%	< 0.001	0.289
**Polynomial regression**		
Linear	< 0.001	< 0.001
Quadratic	< 0.001	< 0.001

^a^Negative control (0.00% 5′-IMP and 3200 kcal ME/kg).

^b^Positive control (0.00% 5′-IMP and 3300 kcal ME/kg).

MDA concentration in the liver of pigs fed the negative control diet was lower (*P* < 0.001) than in pigs fed the positive control diet. However, the negative control diet resulted in a higher (*P* ≤ 0.05) MDA level than 5′-IMP-supplemented diets, except for the diet containing 0.200% 5′-IMP (*P* = 0.666). Pigs fed the positive control diet showed higher liver MDA concentrations (*P* < 0.001) than those supplemented with 5′-IMP, except for those supplemented with 0.200% 5′-IMP (*P* = 0.289). MDA concentration in the liver had a quadratic response (*P* < 0.001) to 5′-IMP supplementation (Fig. 3B). The lowest MDA concentration (1.29 mg/kg) was estimated to be reached with 0.097% 5′-IMP supplementation.

### AST and ALT levels in plasma and creatine levels in plasma and *m. longissimus lumborum*

No differences (*P* > 0.05) were observed in AST, ALT, or creatine levels (in plasma or muscle) between pigs fed negative and positive control diets (Table 5).

**Table 5:** Concentrations of alanine aminotransferase (ALT), aspartate aminotransferase (AST), and creatine in blood plasma, as well as creatine concentration in *longissimus lumborum* muscle of 75–100 kg barrows fed diets containing different levels of inosine-5-monophosphate (5′-IMP) and metabolizable energy (ME).

Item	Blood plasma			Muscle
	ALT, mg/dL	AST, mg/dL	Creatine, mg/dL	Creatine, mg/dL
NC^a^	30.25	38.54	1.36	5.60
PC^b^	30.58	43.39	1.38	5.33
0.050%	37.88	43.66	1.58	5.91
0.100%	36.36	44.70	1.37	6.04
0.150%	36.61	44.43	1.47	6.06
0.200%	36.53	48.65	1.30	6.23
Mean	34.51	43.66	1.41	5.86
SD	5.290	7.190	0.183	0.433
SEM	0.720	0.978	0.020	0.059
**Contrasts**				
NC × PC	0.882	0.191	0.790	0.105
NC × 0.050%	0.003	0.180	0.037	0.066
NC × 0.100%	0.014	0.090	0.929	0.010
NC × 0.150%	0.014	0.124	0.282	0.007
NC × 0.200%	0.010	0.012	0.575	< 0.001
PC × 0.050%	0.004	0.926	0.048	0.001
PC × 0.100%	0.000	0.725	0.856	< 0.001
PC × 0.150%	0.020	0.765	0.376	< 0.001
PC × 0.200%	0.009	0.192	0.381	< 0.001
**Polynomial regression**				
Linear	0.112	0.459	0.186	< 0.001
Quadratic	0.076	0.715	0.142	0.212

^a^Negative control (0.00% 5′-IMP and 3200 kcal ME/kg).

^b^Positive control (0.00% 5′-IMP and 3300 kcal ME/kg).

Plasma ALT was lower (*P* ≤ 0.05) in pigs fed control diets than in pigs supplemented with 5′-IMP. Similarly, plasma AST was lower (*P* = 0.012) in pigs fed the negative control diet than in animals supplemented with 0.200% 5′-IMP, but there were no differences (*P* > 0.05) between the positive control and 5′-IMP-supplemented diets.

**Figure 4:**
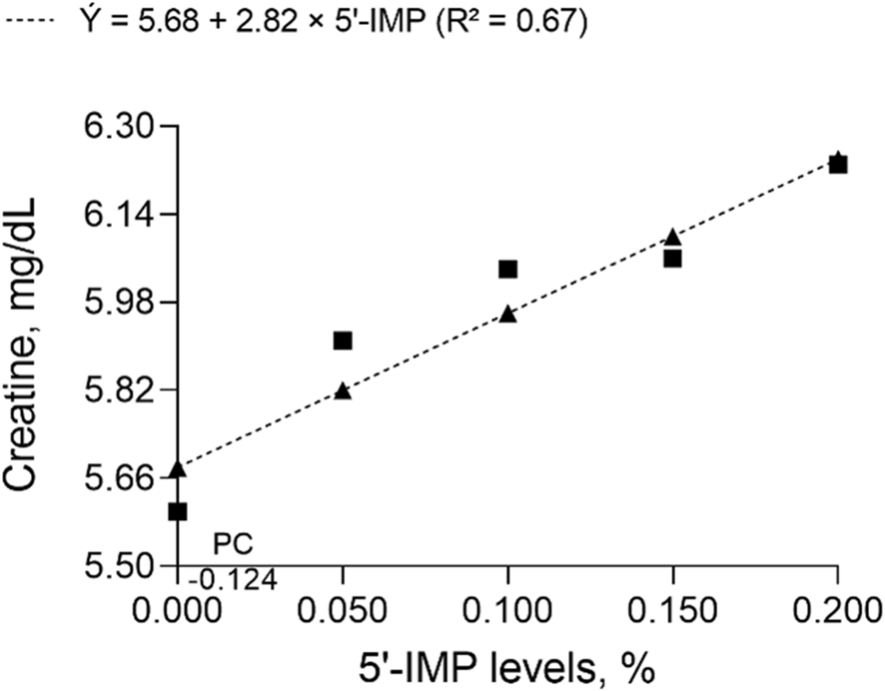
Creatine concentration in the *longissimus lumborum* muscle of 75–100 kg barrows fed diets containing different levels of inosine-5′-monophosphate (5′-IMP) and a 100 kcal/kg lower metabolizable energy level than the positive control (high-energy) diet.

The negative control diet resulted in lower (*P* = 0.037) plasma creatine levels than the diet supplemented with 0.050% 5′-IMP. There were no differences (*P* > 0.05) in plasma ALT between positive control and 5′-IMP-supplemented diets. All levels of 5′-IMP supplementation increased muscle creatine levels compared with control diets (*P* ≤ 0.05). Furthermore, as shown in Fig. 4, muscle creatine increased linearly (*P* < 0.001) with 5′-IMP supplementation level. Thus, the highest muscle creatine concentration was achieved with 0.200% 5′-IMP supplementation.

### Carcass traits

**Figure 5:**
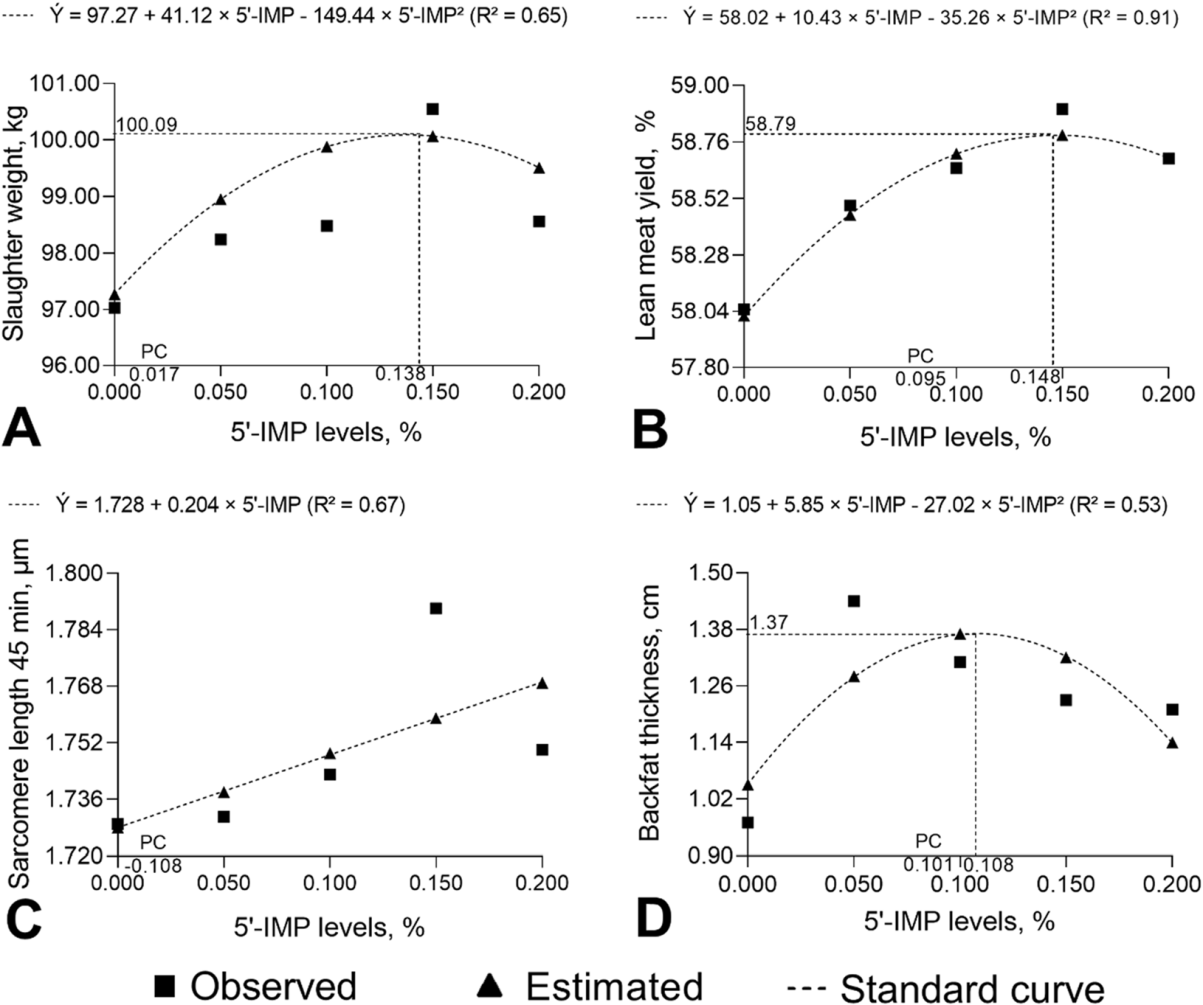
Slaughter weight (**A**), lean meat yield (**B**), sarcomere length 45 min after slaughter (**C**) and backfat thickness (**D**) of 75–100 kg barrows fed diets containing different levels of inosine-5′-monophosphate (5′-IMP) and a 100 kcal/kg lower metabolizable energy level than the positive control (high-energy) diet.

No differences (*P* > 0.05) in slaughter weight were observed between pigs fed negative and positive control diets (Table 3). The slaughter weight of pigs fed negative or positive control diets was lower (*P* = 0.004 and *P* = 0.037, respectively) than that of pigs fed the diet supplemented with 0.150% 5′-IMP. Slaughter weight had a quadratic response (*P* < 0.015) to 5′-IMP supplementation (Fig. 5A). The highest slaughter weight (100.09 kg) was estimated to be reached with 0.138% 5′-IMP supplementation.

The lean meat yield of pigs fed the negative control diet was lower (*P* < 0.001) than that of pigs fed the positive control diet or 5′-IMP-supplemented diets (*P* ≤ 0.05). The positive control diet resulted in a lower (*P* < 0.020) lean meat yield than the 0.150% 5′-IMP-supplemented diet (Table 3). Lean meat yield was shown to have a quadratic relationship with 5′-IMP level (*P* < 0.001): the highest yield (58.79%) was predicted to occur with 0.148% 5′-IMP supplementation (Fig. 5B).

The *m. longissimus lumborum* depth of pigs did not differ significantly (*P* > 0.05) between treatments (Table 3). The backfat thickness of pigs fed the negative control diet was lower (*P* = 0.002) than that of pigs fed the positive control diet or diets supplemented with 0.050%, 0.100%, or 0.150% 5′-IMP (*P* = 0.003, *P* = 0.009, and *P* = 0.025, respectively). There were no differences (*P* > 0.05) in backfat thickness between positive control and 5′-IMP-supplemented diets (Table 3). Furthermore, as shown in Fig. 5D, backfat thickness had a quadratic response (*P* < 0.001) to 5′-IMP supplementation. The highest backfat thickness (1.37 cm) was estimated to be reached with 0.108% 5′-IMP supplementation.

### Sarcomere length in *m. longissimus lumborum*

Sarcomere length in *longissimus lumborum* was measured at 45 min and 24 h after slaughter. Pigs fed the negative control diet showed a higher sarcomere length at 45 min after slaughter (*P* = 0.045) than pigs fed the positive control diet (Table 3). However, the negative control diet resulted in a lower (*P* ≤ 0.05) sarcomere length at 45 min than diets supplemented with 0.150% or 0.200% 5′-IMP. Similarly, sarcomere length was lower in pigs fed the positive control diet (*P* ≤ 0.05) than in animals fed 5′-IMP-supplemented diets. Sarcomere length at 45 min increased linearly (*P* = 0.004) with 5′-IMP supplementation level (Fig. 5C). The highest sarcomere length at 45 min after slaughter was estimated to be reached with 0.200% 5′-IMP supplementation.

At 24 h after slaughter, sarcomere length in *longissimus lumborum* was higher in pigs fed the negative control diet (*P* < 0.001) than in pigs fed the positive control or the 0.200% 5′-IMP-supplemented diet (*P* = 0.025). However, the positive control diet resulted in a lower (*P* ≤ 0.05) sarcomere length at 24 h than 0.050%, 0.100%, and 0.150% 5′-IMP-supplemented diets (Table 3).

## Discussion

The largest de novo biosynthesis site of 5′-IMP is the liver. In this organ, 5′-IMP participates in several biochemical processes, such as ischemia^6^, cell growth and proliferation^7,18^ and regulation of insulin and glucose metabolism^10,40^.

Previous studies have shown that 5′-IMP supplementation influences the relative weight of the liver. In assessing dietary supplementation of newborn rats with NT, Novak et al.^11^ observed a reduction in the relative liver weight of rats fed the NT diet compared with unsupplemented animals. The same effect on the relative weight of the liver was reported in broilers fed diets supplemented with 5′-IMP and allopurinol^12^.

The reduction in the relative weight of the liver in different species corroborates the linear reduction in this parameter with increasing 5′-IMP supplementation levels observed in the current study in finishing pigs. These findings suggest that 5′-IMP supplementation enhances liver functionality and minimizes metabolic disorders that could increase mitochondrial oxidation and apoptosis in hepatic tissues.

Oxidative phosphorylation, the main ATP synthesis pathway, takes place in the electron transport chain located in the inner membrane of mitochondria^1,2^. The process depends on the energy released by oxidation of carbohydrates, lipids, and peptides, which generates a proton gradient throughout the inner mitochondrial membrane, used for ATP synthesis^13^. The respiration rate of liver mitochondria is associated with the rate of ATP synthesis^14^. During oxidative phosphorylation, ROS are generated by complexes I and III, leading to excess production of these substances. Thus, mitochondria and mitochondrial DNA are exposed to oxidation, causing metabolic dysfunction and further increasing ROS synthesis^15,16^.

5′-IMP, a precursor of other nucleotides, can be metabolized to adenosine monophosphate (AMP) or guanosine monophosphate (GMP) and subsequently converted to ATP or GTP, respectively, according to the metabolic needs of the body^17,18^. ATP is the main energy carrier for metabolic activities; various metabolic processes are benefited by high concentrations of ATP^19–21^. GTP is the second most important energy molecule for cellular activities. High GTP concentrations are associated with gene expression and enzymatic activities responsible for the growth and proliferation of immune cells, protein synthesis, and DNA synthesis^22–24^.

5′-IMP supplementation is important in situations of rapid muscle growth and oxidative stress, such as occurs in finishing pigs. Under these conditions, the amount of 5′-IMP synthesized by the body is not sufficient to meet metabolic requirements^7^. We hypothesized that dietary supplementation of an exogenous 5′-IMP source could reduce ATP and GTP consumption in de novo biosynthesis of 5′-IMP (seven molecules of ATP and one of GTP are needed to synthesize one molecule of 5′-IMP) and stimulate the recovery pathway of 5′-IMP and other nucleotides, such as ATP and GTP^25,26^. These effects are relevant, given that intracellular concentrations of ATP and GTP control major metabolic activities and may influence oxidative phosphorylation, ROS-mediated oxidation of mitochondrial DNA, and anabolism.

In this study, oxidative phosphorylation of liver mitochondria was assessed by determining mitochondrial respiration rate, respiratory control, and ADP/O ratio. Supplementation of finishing pig diets with 5′-IMP, particularly at 0.200%, increased state III (after ADP addition) mitochondrial respiration rate in the presence of either substrate (succinate or α-ketoglutarate) compared with positive and negative control diets.

State III respiration rate in succinate medium was influenced by 5′-IMP level: the highest rate (66.77 nmol/mg) was estimated to be achieved by using 0.200% 5′-IMP. Similarly, in the α-ketoglutarate medium, state III respiration rate the highest rate (34.37 nmol/mg) was estimated to be achieved by using 0.200% 5′-IMP, thus 0.200% 5′-IMP supplementation provided the highest liver mitochondrial respiration rate regardless of incubation medium.

The fact that the ADP/O ratio was not altered in state III indicates that there was an increase in the respiratory capacity of liver mitochondria. It is likely that ADP stimulated liver metabolic activity, thereby increasing ATP production from ADP and, consequently, oxygen consumption^27^. Thus, finishing pigs supplemented with 5′-IMP exhibited higher ADP-stimulated mitochondrial respiration efficiency.

Mitochondrial respiration was associated with higher efficiency of oxidative phosphorylation when ADP was present at higher concentrations, that is, in the face of work overload. Such a condition is similar to that occurring in finishing pigs from new genetic lines. The results suggest that dietary supplementation of 5′-IMP might have benefited oxidative phosphorylation, an important route for ATP synthesis.

ROS are formed in mitochondria, mainly in the basal complex and complex III of the electron transport chain^28^. ROS are well known to be involved in oxidative stress and lipid peroxidation^16^. In assessing the antioxidant status in the liver of finishing pigs, we found that diets containing 0.050, 0.100, and 0.150% 5′-IMP were less effective in promoting the removal of free radicals compared with the negative control diet and resulted in lower liver MDA concentrations than negative and positive control diets.

DPPH^•^ scavenging activity and MDA concentration decreased quadratically as a function of 5′-IMP supplementation level. The minimum inflection points were estimated to be reached by supplementation with 0.101% (DPPH^**·**^ activity) and 0.097% 5′-IMP (MDA level). The fact that the lowest values of both variables were estimated at similar 5′-IMP concentrations is interesting, given that a reduction in DPPH^**·**^ scavenging activity is expected to lead to an increase in ROS-mediated lipid peroxidation, affording degradation metabolites such as MDA. The simultaneous decrease in MDA concentration with 5′-IMP supplementation suggests that ROS were formed in smaller amounts during mitochondrial respiration, particularly during state III respiration. Such a reduction in ROS production might be related to the higher liver mitochondrial respiration rate and improved respiratory control, when the ADP is added, i.e., to higher oxygen uptake to convert ADP in ATP, a condition that dissipates the mitochondrial proton gradient, increases the mitochondrial flow of electrons and prevent the ROS generation.

This hypothesis was tested in previous research. Jing et al.^29^ used genetically modified mice lacking the sirtuin-3 gene to investigate oxygen consumption and oxidative stress. The authors reported that genetically modified mice showed lower oxygen consumption and increased oxidative stress. In a similar line of research, Heise et al.^20^ found that ROS production correlated negatively with respiratory control.

The reduction in MDA concentration and DPPH^**·**^ scavenging activity in the liver of finishing pigs may also be partially explained by the fact that mitochondria and mitochondrial DNA are exposed to oxidation by ROS, which are generated by complexes I and III. Supplementation with 5′-IMP might have contributed to antioxidant defense against ROS, as 5′-IMP and other nucleotides act directly in DNA repair and replication^30^.

The higher state III respiration rate and respiratory control of liver mitochondria might be associated with the altered antioxidant status of the liver. We measured plasma concentrations of ALT and AST as indicators of liver functional status^31^. Plasma ALT and AST are tests for the detection of hepatocellular injury in most animal species, but in non-rodents as pigs, ALT and AST also are associated with metabolic adaptation^32^. ALT is more specific and sensitive biomarker than AST for hepatocellular injury, when aminotransferase activities are increased as a consequence of hepatotoxicity the concentration of the ALT increase is usually greater than AST^32^. In the present study, diets 5′-IMP-supplemented showed higher (P ≤ 0.05) plasma ALT concentration than pigs fed with control diets, but the differences between concentrations were too low to be related to hepatocellular damage, even more, that the plasma ALT was lower than AST, indicating a normal liver functionality and possible interaction between plasma ALT with the metabolic adaption to 5′-IMP supplementation in diets of finishing pigs.

Creatine is a metabolite used as an energy substrate for muscle growth^33^. In the current study, plasma creatine levels were higher with 0.050% 5′-IMP supplementation than with the negative control diet. Supplementation of diets with 0.100%, 0.150%, and 0.200% 5′-IMP increased muscle creatine level compared with the negative diet, and all 5′-IMP-supplemented diets enhanced muscle creatine compared with the positive control diet.

Creatine concentration increased linearly with 5′-IMP supplementation level; the highest creatine level was estimated to be reached by supplementation with 0.200% 5′-IMP. These findings indicate that 5′-IMP influenced protein synthesis, promoting an increase in muscle mass and slaughter weight (as observed in pigs supplemented with 0.150% 5′-IMP compared with control diets). Muscle creatine levels are proportional to muscle mass and lean meat yield^34^.

In this study, the increase in muscle creatine concentration might have directly contributed to the increase in lean meat yield in pigs fed 5′-IMP-supplemented diets compared with pigs fed the negative control diet. Furthermore, pigs supplemented with 0.150% 5′-IMP showed a higher lean meat yield than pigs fed the positive control diet, even though the supplemented diet had a 100 kcal ME/kg lower energy level than the positive control diet. These results suggest that 5′-IMP supplementation promotes an increase in energy and protein synthesis. The increase in muscle creatine level also reflected on sarcomere length at 45 min after slaughter. Pigs fed diets supplemented with 0.150 and 0.200% 5′-IMP had higher sarcomere lengths than pigs fed negative or positive control diets.

The increase in muscle and plasma creatine levels can be explained by the activity of 5′-IMP on the mTOR complex. A previous study showed that 5′-IMP and other purines acted on this complex, stimulating anabolism^9,18^. Another possible explanation is the improvement in oxidative phosphorylation efficiency promoted by supplementation with 0.200% 5′-IMP, which might have increased creatine availability for metabolic pathways.

In this study, we evaluated the following quantitative carcass and *m. longissimus lumborum* quality parameters: slaughter weight, lean meat yield, muscle depth, sarcomere length in *m. longissimus lumborum* at 45 min and 24 h after slaughter, and backfat thickness. Lean meat yield is the main and most practical quantitative carcass parameter; it is an important index to assess the amount of lean meat in carcasses, contributing to the development of new swine genotypes^35^. *M. longissimus lumborum* is one of the most appreciated cuts by consumers, having high commercial value. Backfat thickness is measured in the P2 region and represents the amount of fat between *m. longissimus lumborum* and the skin. Sarcomere length in *longissimus lumborum* has been associated with muscle tenderness in several studies^36–38^. The shorter the sarcomere length, the less tender the meat, a characteristic that indicates low muscle quality.

In comparing negative and positive control diets, we observed that, although there were no differences in slaughter weight, the lean meat yield of pigs fed the positive control diet was higher than that of pigs fed the negative control diet. This finding demonstrated that the lower ME level of the negative control diet negatively influenced carcass lean meat deposition. The negative control diet (3200 kcal ME/kg) resulted in longer sarcomere lengths at 45 min and 24 h after slaughter compared with the positive control diet (3300 kcal ME/kg); however, the negative control diet afforded a lower backfat thickness than the positive control diet. The positive control diet, which had a 100 kcal ME/kg higher energy level, provided greater energy input, part of which was used to increase protein and muscle deposition, thereby increasing lean meat yield. Surplus energy was stored in the form of lipids in adipocytes, promoting an increase in backfat thickness.

5′-IMP-supplemented diets had a lower level of ME (3200 kcal ME/kg). Pigs fed the 0.150% 5′-IMP-supplemented diet had the highest slaughter weight, higher than that of pigs fed control diets. Slaughter weight showed a quadratic response to 5′-IMP level; the highest yield (100.09 kg) was estimated to be reached with 0.138% 5′-IMP. Similarly, pigs fed 5′-IMP-supplemented diets had a higher lean meat yield than pigs fed the negative control diet, and pigs supplemented with 0.150% 5′-IMP had higher lean meat yield than pigs fed the positive control diet (3300 kcal ME/kg). Lean meat yield showed a quadratic response to 5′-IMP level; the highest yield (58.79%) was estimated to be reached with 0.148% 5′-IMP.

The higher slaughter weight and lean meat yield of pigs supplemented with 0.150% 5′-IMP can be explained by the increase in muscle creatine concentration promoted by supplementation. Another possible explanation is the conversion of 5′-IMP to ATP and GTP, the main energy sources for cellular activities, influencing protein synthesis through the action of ATP and ATPases^6,7^ or by the relationship between GTP and the mTORC complex^19^.

Sarcomere length at 45 min after slaughter was higher in pigs supplemented with 0.150% and 0.200% 5′-IMP than in pigs fed the negative control diet. Similarly, the parameter was higher in pigs fed 5′-IMP-supplemented diets than in pigs fed the positive control diet. A diet containing 0.200% 5′-IMP was estimated to result in the longest sarcomere length (1.769 µm). Similarly, sarcomere length at 24 h after slaughter increased with 0.050%, 0.100%, and 0.150% 5′-IMP supplementation compared with the positive control diet.

The greater sarcomere lengths at 45 min and 24 h after slaughter in pigs supplemented with 0.150% and 0.200% 5′-IMP can be explained in part by the higher muscle creatine concentration proportionated by 5′-IMP supplementation. The highest availability of creatine may stimulates muscle growth, which, during postmortem aging, increases the availability of energetic substrates, thereby prolonging the time until ATP begins to be consumed.

It is also known that 5′-IMP participates in the dissociation of the actin–myosin complex during the first 24 h of postmortem aging, an effect previously observed in processed^39^ and fresh^40^ pork meat. According to Miller^41^, the overlap between actin and myosin is smaller in longer sarcomeres, resulting in reduced resistance when cutting the fibers of the *m. longissimus lumborum*.

Backfat thickness results showed that dietary 5′-IMP supplementation combined with low ME level provided an increase in energy input, given that pigs supplemented with 0.050%, 0.100%, and 0.150% 5′-IMP showed greater backfat thickness than pigs fed the negative control diet. Such a greater energy input was evidenced by the lack of differences in backfat thickness between pigs fed 5′-IMP diets and animals fed the positive control diet, which had a higher energy level (3200 vs 3300 kcal ME/kg). Furthermore, we observed a quadratic response in backfat thickness with 5′-IMP level supplementation. The increase in energy input with 5′-IMP supplementation might be associated with the conversion of 5′-IMP to ATP and GTP, the main energetic molecules for cellular activities^6,7^. The reduction in backfat thickness after the supplementation with 0.108% 5′-IMP demonstrated by the quadratic response, on the other hand, might have occurred as a result of the activity of IMP dehydrogenase (IMPDH) in lipid deposition.

According to Whitehead et al.^42^, insulin stimulates phosphorylation and translocation of IMPDH to adipose tissues; both processes are blocked by inhibition of the insulin substrate receptor phosphatidylinositol 3-kinase. Oleic acids stimulate IMPDH translocation only, and inhibition translocation of IMPDH to adipose tissues results in lower amounts of lipids. Thus, the authors concluded that IMPDH exerts regulatory and dynamic roles in lipid deposition and fatty acid metabolism.

The results of this study demonstrated that dietary 5′-IMP supplementation promoted benefits to oxidative phosphorylation by increasing mitochondrial respiration rate without altering the ADP/O ratio. Demonstrating that with the liver metabolic activity stimulated, process in which the energy is required to supply an increased metabolism, the animals supplemented with 5′-IMP presented a higher efficiency of mitochondrial oxidative phosphorylation to support the energy requirement. Supplementation also reduced liver tissue oxidation and increased plasma and muscle creatine levels, slaughter weight, lean meat yield, sarcomere length at 45 min and 24 h after slaughter, and backfat thickness in finishing barrows.

The influence of 5′-IMP supplementation on slaughter weight, lean meat yield and backfat thickness as compared with negative and positive control diets allowed us to infer that dietary 5′-IMP supplementation increased energy availability and protein synthesis through the possible use of 5′-IMP as an energy and protein additive. Further studies are needed to identify the mechanisms by which 5′-IMP produced these effects.

## Conclusion

Supplementation of finishing pig diets with different levels of 5′-IMP increased the respiratory efficiency of liver mitochondria, reduced lipid peroxidation, and enhanced muscle creatine levels, slaughter weight, lean meat yield, sarcomere length at 45 min and 24 h after slaughter, and backfat thickness, demonstrating that 5′-IMP supplementation contributes to several metabolic processes, mainly those of energy and protein synthesis.

## Material and methods

### Animal ethics statement

The experiment was conducted at the Pig Farming Section of the Iguatemi Experimental Farm, State University of Maringá, Brazil. All animal experimental procedures were approved by the Animal Ethics Committee of the State University of Maringá (protocol No. 9056170220). Animal care and use standards were based on the National Council for the Control of Animal Experimentation (https://antigo.mctic.gov.br/mctic/opencms/institucional/concea/paginas/legislação.html). Study design, animal experiments, and reporting followed the ARRIVE guidelines (https://arriveguidelines.org/arrive-guidelines).

### Facilities, animals, and experimental design

Pigs were housed in a barn covered with fiber cement tiles and divided into 40 pens (1.88 m^2^ each) with cement floor. Each pen was equipped with a semi-automatic feeder at the front and a nipple drinker at the back. The animals had ad libitum access to water and feed throughout the experimental period.

A total of 54 castrated male pigs (mean initial weight of 75.62 ± 0.96 kg and mean final weight of 102.26 ± 3.23 kg) were distributed in a randomized complete block design throughout the trial period (75 to 100 kg) with nine blocks and six treatments. Each block the animal are randomly assigned to the treatment and was considered an experimental unit.

### Diet

Pigs were fed one of the following six experimental diets: a positive control diet containing 3300 kcal metabolizable energy (ME)/kg, a negative control diet containing 3200 kcal ME/kg, or the negative control diet supplemented with 0.050, 0.100, 0.150, or 0.200% 5′-IMP. Experimental diets were composed of corn, soybean meal, minerals, vitamins, and additives (Table 6) and were formulated to meet the nutritional requirements of pigs according to National Research Council guidelines^43^, except for ME.

**Table 6:** Composition of experimental diets.

Ingredients (%)	NC^e^	5′-IMP^a^ (%)				PC^f^
		0.050	0.100	0.150	0.200	
Corn	79.12	79.12	79.12	79.12	79.12	80.49
Soybean meal	16.82	16.82	16.82	16.82	16.82	16.60
Soybean oil	–	–	–	–	–	0.750
Dicalcium phosphate	0.598	0.598	0.598	0.598	0.598	0.596
Limestone	0.744	0.744	0.744	0.744	0.744	0.747
Salt	0.225	0.225	0.225	0.225	0.225	0.223
5′-IMP	–	0.050	0.100	0.150	0.200	–
Inert^b^	1.900	1.850	1.800	1.750	1.700	–
Vitamin and mineral supplement^c^	0.400	0.400	0.400	0.400	0.400	0.400
l-Lysine HCl 78.4%	0.160	0.160	0.160	0.160	0.160	0.164
Enramycin	0.020	0.020	0.020	0.020	0.020	0.020
Feed dry^d^	0.015	0.015	0.015	0.015	0.015	0.015
**Calculated composition, %**						
Metabolizable energy (Mcal/kg)	3,200	3,200	3,200	3,200	3,200	3,300
Crude protein	14.00	14.00	14.00	14.00	14.00	14.00
Total calcium	0.500	0.500	0.500	0.500	0.500	0.500
Available phosphorus	0.190	0.190	0.190	0.190	0.190	0.190
Potassium	0.561	0.561	0.561	0.561	0.561	0.561
Sodium	0.100	0.100	0.100	0.100	0.100	0.100
Chlorine	0.248	0.248	0.248	0.248	0.248	0.249
SID lysine	0.690	0.690	0.690	0.690	0.690	0.690
SID methionine	0.200	0.200	0.200	0.200	0.200	0.200
SID methionine + cysteine	0.425	0.425	0.425	0.425	0.425	0.425
SID threonine	0.449	0.449	0.449	0.449	0.449	0.449
SID tryptophan	0.135	0.135	0.135	0.135	0.135	0.135
SID valine	0.627	0.627	0.627	0.627	0.627	0.627
SID leucine	1.197	1.197	1.197	1.197	1.197	1.197
SID isoleucine	0.502	0.502	0.502	0.502	0.502	0.502
SID arginine	0.791	0.791	0.791	0.791	0.791	0.791
SID histidine	0.350	0.350	0.350	0.350	0.350	0.350
SID phenylalanine	0.616	0.616	0.616	0.616	0.616	0.616
SID phenylalanine + tyrosine	1.072	1.072	1.072	1.072	1.072	1.072

^a^Inosine-5′-monophosphate.

^b^Kaolinite.

^c^Provided per kilogram: vitamin A, 30,000 UI; vitamin D3, 5000 UI; vitamin E, 120 UI; vitamin K, 5 mg; vitamin B12, 120 mcg; niacin, 150 mg; calcium pantothenate, 75 mg; folic acid, 8 mg; choline chloride, 0.48 g; iron, 350 mg; copper, 15 mg; manganese, 250 mg; zinc, 0.75 g; iodine, 10 mg; selenium, 3 mg.

^d^Antioxidant.

^e^Negative control (0.00% 5′-IMP and 3200 kcal ME/kg).

^f^Positive control (0.00% 5′-IMP and 3300 kcal ME/kg).

### Slaughter procedures

At the end of the experiment, pigs were fasted for 24 h and weighed to obtain the slaughter weight. Slaughter was carried out at the slaughterhouse of the Iguatemi Experimental Farm. The animals were slaughtered by exsanguination after electrical stunning (200 W). Pig carcasses were scalded in water (60 °C), dehaired, singed, washed, eviscerated, split into two, weighed, and stored in a cold room (0.5 ± 1.0 °C) for 24 h.

### Relative weight of the liver

Following evisceration, nine livers per treatment (*n* = 56) were weighed and used to calculate the relative weight of the liver by the following equation: Relative weight of the liver = Liver weight × 100/Body weight after fasting.

### Collection of liver samples

After slaughter, specimens (15 g) from the medial segment of the liver were excised from six pigs per treatment (*n* = 36) and promptly analyzed for mitochondrial respiration. For determination of oxidative stress, specimens (15 g) from the medial segment of the liver were collected from nine pigs per treatment (*n* = 54), frozen at − 20 °C until the end of the slaughter process, and taken to the laboratory for extraction and analysis.

### Isolation of liver mitochondria

Liver specimens were immediately immersed in cold buffer containing 200 mM mannitol, 75 mM sucrose, 0.2 mM ethylene glycol tetraacetic acid, 2 mM tris(hydroxymethyl)aminomethane hydrochloride (Tris–HCl, pH 7.4), and 50 mg bovine serum albumin. A Dounce-type homogenizer was used to lyse cells, and mitochondria were isolated by differential centrifugation^44^.

### Mitochondrial respiratory activity

Mitochondrial oxygen consumption was measured polarographically by using a Teflon-coated platinum electrode^44^. Mitochondria were incubated in a closed-chamber oxygraph in medium (2.0 mL) containing 0.25 M mannitol, 5 mM sodium diphosphate, 10 mM KCl, 0.2 mM EDTA, and 10 mM Tris–HCl (pH 7.4). Succinate and α-ketoglutarate (both at 10 mM) were used as electron donor substrates for complexes I and II, respectively, of the mitochondrial electron transport chain. ADP (final concentration of 0.125 mM) was added at predetermined times. Oxygen consumption rates were calculated from the slope of oxygen consumption plots generated on paper by the recording system. Results are expressed in nmol min^−1^ mg^−1^ protein. Oxygen consumption was measured under three conditions: (i) before ADP addition (basal or substrate respiration), (ii) shortly after ADP addition (state III respiration), and (iii) after cessation of ADP stimulation (state IV respiration). Respiratory control was calculated as the ratio of oxygen consumption in state III to state IV. The ADP/O ratio was determined as described by Chance and Williams^45^.

### Sample preparation for antioxidant analysis

For antioxidant analysis by DPPH^**·**^ assay, liver samples (5 g) were mixed with methanol (15 mL), homogenized with an Ultra Turrax for 1 min, and filtered through qualitative filter paper No. 42. For the thiobarbituric acid-reactive substances (TBARS) assay, liver samples (5 g) were mixed with 15 mL of extraction solution (7.5% trichloroacetic acid, 0.1% gallic acid, and 0.1% EDTA), homogenized with an Ultra Turrax for 1 min, and filtered through qualitative filter paper No. 42. Supernatants (deproteinized liver tissues) were stored in Falcon tubes at − 20 °C until use^46^.

### DPPH assay

The ability of liver tissues to scavenge DPPH^•^ (D9132, Sigma-Aldrich, St. Louis, MI, USA) was determined according to the method described by Brand-Williams et al.^47^. Deproteinized liver tissue samples (200 µL) were homogenized with 1.8 mL of DPPH^•^ solution (0.0024 g of DPPH^•^ in 100 mL of 96.5% methanol) and incubated in the dark for 30 min. The absorbance was measured at 515 nm on a spectrophotometer (Sp 22, Biospectro, Curitiba, PR, Brazil).

### TBARS assay

MDA concentrations were determined by the TBARS method^46^. Deproteinized liver tissue samples (500 µL) were homogenized with 2.0 mL of a solution consisting of 15% thiobarbituric acid, 10% trichloroacetic acid, and 0.06% HCl. Subsequently, the mixtures were incubated in a water bath at 100 °C for 15 min and allowed to cool for 5 min. Absorbance was determined spectrophotometrically (SP 22, Biospectro, Curitiba, PR, Brazil) at 532 nm.

### AST and ALT levels in plasma and creatine levels in plasma and m. *longissimus lumborum*

Pigs weighing on average 100 kg live weight (*n* = 54) were fasted for 6 h before sample collection. Blood samples were collected from the jugular vein into tubes containing EDTA and centrifuged at 3000×*g* for 15 min. The plasma was withdrawn with an automatic pipette and added to Eppendorf tubes. Plasma levels of AST, ALT, and creatine were determined by using test kits (Gold Analisa, Belo Horizonte, MG, Brazil). All laboratory procedures were performed according to kit instructions.

After slaughter of pigs, 15 g of m. *longissimus lumborum* was excised from each animal and stored on ice until extraction at the laboratory. Creatine extraction was performed as proposed by Chamruspollert et al.^48^. Creatine concentrations were measured by reading the absorbance (SP 22, Biospectro, Curitiba, PR, Brazil) of the resulting supernatants at 450 nm.

### Carcass traits

Carcasses were chilled (0–1 °C) for 24 h and then subjected to quantitative evaluation, according to the Brazilian Method of Swine Carcass Classification^49^. Carcass traits were evaluated by measuring slaughter weight, lean meat yield (LMY, %), *longissimus lumborum* depth (LLD, mm), and backfat thickness (BF, mm). BF and LLD were measured between the last thoracic vertebra and the first lumbar vertebra, 6 cm away from the vertebral column, using a digital caliper (precision of 0.02 mm; Digimess, King tools, Sheffield, England) after 24 h postmortem. Carcass LMY was determined using the equation proposed by Irgang et al.^50^, as follows: LMY = 60 − [(BF × 0.58) + (LLD × 0.10)].

### Sarcomere length in *m. longissimus lumborum*

The method for determining sarcomere length was similar to that previously described by Cross, West, and Dutson^51^. Samples of *longissimus lumborum*, located between the last thoracic vertebra and the first lumbar vertebra, were collected from eight animals per treatment at two different periods (45 min and 24 h after slaughter).

To measure sarcomere length, we collected and fixed chilled samples of *longissimus lumborum* in 10% buffered formalin (pH 7.0–7.2) for 24 h. Subsequently, samples were processed for paraffin embedding. Semi-serial 6 µm thick longitudinal histological sections were obtained using a microtome. The cuts were distended in a histological water bath at 45 °C and transferred to slides. Slides were placed on a wood support and incubated at 60 °C for 24 h to allow for greater adherence between the paraffin and the slide. After deparaffinization in an oven at 60 °C, samples were washed in running water for 2 min, treated with an aqueous solution of 0.25% potassium permanganate for 10 min, and then washed in running water for another 3 min. Subsequently, samples were immersed in oxalic acid for 5 min, washed in running water for 3 min, and stained with 10% Mallory’s phosphotungstic acid-hematoxylin 0.5 g of hematoxylin, and 1 mL of hydrogen peroxide in a total volume of 500 mL for 24 h. The stain was placed in an amber vial and stabilized for 2 to 3 days before use.

Sections were analyzed under an optical microscope (Olympus BX40 equipped with a Nikon DS-Fi1 camera connected to a Nikon Digital Mira DS-43, Tokyo, Japan) and photographed using an attached camera with oil immersion lenses (100 × objective and 10 × eyepiece). Processing of images, scales, and measurements was performed using Nikon Elements software version 3.22 (https://www.nikon.com/products/microscope-solutions/support/download/software/imgsfw/nis-f_v4600064.htm).

Eight histological slides were obtained per treatment and period (45 min and 24 h after slaughter), using three histological sections per slide. The average sarcomere length (µm) was calculated by determining the length (µm) of 10 sarcomeres in the histological section of each slide, totaling 30 distinct fibers chosen at random per treatment, resulting in a total of 300 observations per animal and period.

### Statistical analyses

The OUTLIER procedure of SAS version 9.0 (Cary, NC, USA, available from https://www.sas.com/en_us/software/on-demand-for-academics.html)^52^ was applied to detect the presence of outliers. Subsequently, data on 5′-IMP levels were subjected to analysis of variance; block effects and 5′-IMP level effects were included in the model. For regression analysis, the degrees of freedom of 0.0, 0.050, 0.100, 0.150 and 0.200% 5′-IMP levels were partitioned in orthogonal polynomials with the PROC MIXED procedure of SAS. Then, a linear response plateau model was used to assess associations with a quadratic model. Comparisons between positive and negative control diets and 5′-IMP levels were performed by Tukey's test using orthogonal contrast analysis (PROC GLM procedure of SAS).
